# Learning Styles of Medical Students, Surgical Residents, Medical Staff, and General Surgery Teachers When Learning Surgery: Protocol for a Scoping Review

**DOI:** 10.2196/57229

**Published:** 2024-10-11

**Authors:** Gabriela Gouvea Silva, Carlos Dario da Silva Costa, Bruno Cardoso Gonçalves, Luiz Vianney Cidrão Nunes, Emerson Roberto dos Santos, Sonia Maria Maciel Lopes, Alba Regina de Abreu Lima, Vânia Maria Sabadoto Brienze, Thaís Santana Gastardelo Bizotto, Júlio César André

**Affiliations:** 1 Center for Studies and Development of Health Education Faculdade de Medicina de São José do Rio Preto São José do Rio Preto Brazil

**Keywords:** general surgery, medical staff, students, medical faculty, medical student, surgery, surgical resident, internship, clinician, teaching method, residency program, residency, training, leaning style, health care education, Kolb, Kolbs model, experiential learning, Felder and Silverman

## Abstract

**Background:**

Learning styles are biological and developmental configurations of personal characteristics that make the same teaching method effective for some and ineffective for others. Studies support a relationship between learning style and career choices in medicine, resulting in learning style patterns being observed in different residency programs, including in general surgery, from medical school to the last stages of training. The methodologies, populations, and contexts of the few studies pertinent to the matter are very different from one another, and a scoping review on this theme will enhance and organize what is already known.

**Objective:**

The goal of this study is to identify and map out data from studies on the learning styles of medical students, surgical residents, medical staff, and surgical teachers.

**Methods:**

The review will consider studies on the learning styles of medical students in a clinical cycle or internship, surgical residents with no restriction on year of residency, medical staff in general surgery, or general surgery’s medical faculty. Primary studies published in English, with no specific time frame, will be considered. The search will be carried out in four databases, and reference lists will be searched for additional studies. Duplicates will be removed, and two independent reviewers will screen the titles, abstracts, and full texts of the selected studies. Data collection will be performed using a tool developed by the researchers. A results summary will be presented with figures, narratives, and tables. A quantitative and qualitative analysis will be carried out and further results will be shared.

**Results:**

The search was funded on September 25, 2023. Data collection was performed in the two following months. Of the 213 articles found, 135 were excluded due to duplication. The remaining 78 articles will have their titles and abstracts analyzed by three of the researchers independently to select those that meet the eligibility criteria. This data is expected to be published in the first semester of 2025.

**Conclusions:**

Conducting a scoping review is the best way to map what is known about a subject. Understanding how students, residents, staff, and even teachers prefer to learn surgery is key to staying up to date and knowing how to best educate those pursuing a surgical career.

**Trial Registration:**

Open Science Framework 75ku4; https://osf.io/75ku4

**International Registered Report Identifier (IRRID):**

DERR1-10.2196/57229

## Introduction

The concept of learning styles first developed at the beginning of 1960 with an increased interest in the individual [1]. According to Dunn [2], everyone has a peculiar learning style, like a signature, so tailoring teaching to different learning styles may help improve results in education.

In the current literature, there are different models to determine learning styles, especially in health care education: Kolb’s model, Felder and Silverman’s model, and Gregorc’s model. Whether learning styles are fixed or flexible and to what extent learning styles can be determined by the context is still being debated today [3].

Kolb [4] describes learning as a process where knowledge is transformed through experience and says that acknowledgment is the combination of appropriation and the transformation of experience. The theory is a holistic model called “experiential learning” and emphasizes the central role experience plays, differing it from other theories [5]. Kolb’s [4] scheme hypothesizes that the learner has a concrete experience that he reflects on. Through reflection, it is possible to formulate abstract concepts and make appropriate generalizations, and then consolidate the understanding by testing the implications of the knowledge in new situations, providing a concrete experience, and the cycle continues. Learners with different learning preferences will have different strengths and weaknesses in the quadrants of the (Kolb) cycle [4]. Based on that, he created the Learning Style Inventory to determine and assess different learning styles [6].

Felder and Silverman [7] created the Index of Learning Survey, which was initially for engineering education but is also valid among medical students. The Index of Learning Survey classifies individuals into four fields: preferred information observation (sensory or intuitive; visual or verbal), active versus reflective information processing, and sequential versus global progression for understanding information [8].

In medical education, it is particularly important to remember that some programs count on learners who have already completed a university degree, and in others, the students have only completed secondary school. Medical education includes postgraduate students and those who are continuing professional development. Each of them will have variable individual constraints, experiences, and preferences [9].

Perry [10] noted that students change their learning approach as they progress over their college years. Students often begin with a “duality” approach, with a clear view between right and wrong, and move toward “multiplicity,” where they recognize that context is important and that there are various valuable sources of knowledge and experience.

Knowledge is the main domain of medical education, but the outcome depends strongly on other domains such as attitude, lifelong learning, empathy, communication, ethics, and professionalism. The clinical environment is challenging for both the student and the teacher, without even mentioning the patient. It is vital to use different learning theories to promote effective learning [9].

Contemporary surgical trainees come from diverse educational, cultural, ethnic, and gender backgrounds [11], and are pressured to develop skills not only as medical experts but also as professionals, scholars, health advocates, managers, collaborators, and communicators [12].

Educating surgeons is an ancient tradition that has existed since the development of surgery [13], and for centuries, surgical residency curricula have been guided primarily by tradition. The apprenticeship model has been one of the essential components of surgical training. It generally involves three steps: assisting at operations, performing operations with expert assistance, and operating without assistance. In modern times, however, there are more complex procedures, performed more regularly and in safer manners, demanding even more prepared professionals [14].

Modern surgical education has been revolutionized by exponents such as Halsted. The historical model of apprenticeship was transformed into the current organized system that we call residency [11].

This scoping review aims to identify and map out data from studies that report the learning styles of medical students, surgical residents, medical staff, and general surgery teachers while learning surgery.

## Methods

### Overview

The proposed scoping review will be carried out according to Arksey and O’Malley’s [15] structure using the first 5 stages: (1) identify the research question; (2) identify relevant studies; (3) select studies; (4) map out the data; and (5) collate, summarize, and report the results. Since this is preliminary research, more studies on the theme will likely be included. Although the sixth stage of Arksey and O’Malley’s structure (consulting) will not be completed in this review, our results can inform this stage in a future study. This structure is also congruent with the Joanna Briggs Institute’s (JBI) scoping review methodology [16].

### Research Question

The research question was elaborated on according to the objective of the review and through the PCC (Population, Concept, Context) model:

- Population: general surgery residents, general surgery, medical students
- Concept: learning style
- Context: surgical education

Therefore, the following research question was proposed. How do learning styles characterize medical students, surgical residents, medical staff, and general surgery teachers while learning surgery?

### Inclusion Criteria

After a discussion involving the researchers, the eligibility criteria were defined.

- Participants: Studies with medical students in the clinical cycle or an internship, surgical residents with no restriction on year of residency, medical staff in general surgery, or general surgery’s medical faculty.
- Concept: The included studies approached “learning styles” of the target population regardless of the chosen instrument to define it.
- Context: The eligible studies were those related to the teaching of the population in question, focusing on surgical education in any country.

### Types of Sources

The following were included in the review: studies with qualitative and quantitative approaches, primary studies, systematic reviews, meta-analyses or meta-syntheses, books, and guidelines published in indexed sources.

### Exclusion Criteria

The review excludes the publications of opinions, consensus, retractions, editorials, websites, and advertisements published in the media. Other exclusion criteria are not being published in indexed sources and gray literature. Publications made in other languages than English were excluded due to translation barriers. Studies that did not focus on learning styles were excluded, as well as those outside medical education in a surgical environment.

The inclusion and exclusion criteria are shown in Textbox 1.

**Inclusion and exclusion criteria.**

**Article type**

Inclusion criteria

- Qualitative approaches
- Quantitative approaches
- Primary studies
- Systematic reviews
- Meta-analyses or syntheses
- Books published in indexed sources
- Guidelines published in indexed sources

Exclusion criteria

- Opinions
- Consensus
- Retractions
- Editorials
- Websites
- Advertisements
- Gray literature

**Language**

Inclusion criteria

- English

Exclusion criteria

- Other languages

### Research Strategy

The search strategy was performed on September 25, 2023, by a librarian who is a digital search strategy expert using 3 descriptors: learning, style, and surgery. There was no time frame restriction in the search. After the research question was created, the keywords were used to identify articles referring to the topic, namely “learning style” and “surgery.” For the combination of descriptors, the Boolean operators “AND” and “OR” were considered. Words were reduced to their root to include variations in writing and broaden the search scope.

The Embase, SCOPUS, Web of Science, and PubMed databases were searched using the descriptors and their synonyms, according to Medical Subject Headings (MeSH), to every strategy term. These databases were selected because they are comprehensive and have a broad coverage of health publications. Articles found in the database searches were tabulated in an Excel Ink 2021 (Microsoft Corporation) spreadsheet.

### Study and Source of Evidence Selection

Of the 213 articles found, 135 were excluded due to duplication. The remaining 78 articles will had their titles and abstracts analyzed by three of the researchers independently to select those that meet the eligibility criteria. Articles that do not mention the eligibility criteria described above will be excluded. In case of divergence, a fourth researcher will be consulted and will give the final opinion about the relevance of the study in answering the research question. Additional sources can be included in the review after a manual search is performed by the researchers if they meet the eligibility criteria, are important to complete the study, and have not been identified by the search strategy.

To align the eligibility criteria among the researchers, the title and abstract of 25 random articles were analyzed by three of the researchers. There was 100% agreement concerning the inclusion and exclusion of the articles. Disagreements regarding the inclusion or exclusion of the articles were discussed until a consensus was reached.

The complete texts of the selected articles will be evaluated by the main researcher based on the eligibility criteria. The reasons for the exclusion of articles that are fully read will be registered and reported in the scoping review. Any disagreement that emerges among the researchers at any stage of the selection process will be solved through discussion or the addition of other researchers. A PRISMA-ScR (Preferred Reporting Items for Systematic Reviews and Meta-Analyses Extension for Scoping Reviews) flow diagram [16] will be used to present the search results and the studies included in the scoping review.

### Data Extraction

After article selection, a form for data extraction will be used to extract data from the full articles. The extracted data will include specific details, shown in Textbox 2. It can be modified and revised as needed during data extraction. The modifications will be described in the scoping review. Any disagreement that emerges among the researchers will be solved through discussion or inclusion of additional researchers. If appropriate, the articles’ authors will be contacted and asked about missing or complementary data when necessary.

**Data extracted from studies.**

**Identification of the article**

- Journal
- Year
- Title
- Author
- Country
- Type of study

**Eligibility/exclusion criteria**

- Participants
- Concept
- Context
- Exclusion

**Details of the article**

- Learning theory
- Learning style instrument
- Impact

### Registration

The present protocol was registered in the Open Science Framework database [17].

### Data Analysis and Presentation

The results will be analyzed considering the study’s objectives and will be presented graphically or in the form of tables. This information will be enriched by a descriptive text that will show, as clearly as possible, how the results are related to the research question. All the researchers will participate in this stage.

## Results

The search was funded on September 25, 2023. Data collection was performed in the two following months. Of the 213 articles found, 135 were excluded due to duplication. The remaining 78 articles had their titles and abstracts analyzed by three of the researchers independently to select those that meet the eligibility criteria. The pool of literature found corresponds to 78 articles that discuss learning styles in surgical environments, from university to faculty. This data is expected to be published in the first semester of 2025.

## Discussion

### Expected Findings

This scoping review aims to map out data from studies that report on the learning styles of medical students, surgical residents, medical staff, and general surgery teachers while learning surgery. We seek to compare results across time, countries, and learning theories presented in the existing literature.

Conducting a scoping review will provide an overview of learning styles in a surgical educational environment and determine gaps in the published literature about this subject. By looking at the teaching-learning process in surgery, we can better understand and guide future medical education.

From the students to the teachers, new pathways to surgical education can be developed and, ultimately, provide better care for patients undergoing surgery.

### Comparison With Prior Work

Previous studies have supported the relationship between learning style and career choice in medicine, resulting in learning style patterns being observed in distinct residency programs, including general surgery, from medical school to the last stages of training. Based on Kolb’s Learning Style Inventory, students classified as accommodating and diverging frequently chose surgery as their career choice, whereas those with a convergent learning style chose internal medicine and those who were assimilators chose academic medicine [18].

However, despite the theme’s relevance, in a preliminary search in the MEDLINE, Cochrane Database of Systematic Reviews, and JBI Evidence Synthesis databases, no scoping reviews were found. Moreover, the methodologies, populations, and contexts of the few pertinent studies are different from one another, and a scoping review on this theme would enhance and organize what is already known.

### Limitations

The main limitation of this protocol is the fact that only English articles were searched, which diminishes the results in terms of language and culture, considering that surgical residency uses different models and resources throughout the world.

### Conclusions

There are several ways to address adult education, with multiple theories, which can superpose, and multiple ways of evaluating learning according to the chosen theory. In health care, several specificities make the process even more complex, since there are a lot of abilities and competencies faced in a short period. Medical science is evolving faster all the time, adding challenges to the education and preparation of professionals.

Surgery education requires developing skills and training in high-risk procedures. How to deal with the students, residents, and surgical staffs’ education, while taking into account their necessities, the new ways of teaching and sharing knowledge, and the speed of scientific knowledge, is key. Knowing a bit more about how these populations learn is vital to providing good education and, ultimately, good care.

## Appendix

Multimedia Appendix 1 Studies found in search.

## Abbreviations

JBI: Joanna Briggs Institute
MeSH: Medical Subject Headings
PCC: Population, Concept, Context
PRISMA-ScR: Preferred Reporting Items for Systematic Reviews and Meta-Analyses Extension for Scoping Reviews

## Data Availability

The data concerning the total papers and the inclusion of papers is available for further analysis in Multimedia Appendix 1.
